# Optimal Subsidy Support for Market-Oriented Transformation of Elderly Care: Focus on the Gap between Supply and Demand in Aging Regions of China

**DOI:** 10.3390/healthcare8040441

**Published:** 2020-10-29

**Authors:** Huan Song, Sihang Yu, Feng Liu, Xuan Sun, Tao Sun

**Affiliations:** 1 Zhou Enlai School of Government, Nankai University, Tianjin 300350, China; songhuan0108@126.com (H.S.); yusihangchn@163.com (S.Y.); sunxuan@nankai.edu.cn (X.S.); 2 School of Public Finance and Administration, Tianjin University of Finance & Economics, Tianjin 300222, China; liufeng@tjufe.edu.cn

**Keywords:** elderly care, market-oriented transformation, optimal subsidy, fiscal support, social welfare, Stackelberg game

## Abstract

Satisfying the growing care demands of the elderly has become a major policy issue under the trend of rapidly aging of the population, especially in developing countries. Although the market-oriented transformation on the supply side is a sustainable way to cope with the pressing demands of elderly care in the long term, the conflict between private and public interests seriously impedes the transformation process in its early stage. From the perspective of maximizing social welfare, this study took the specific situation of China as an example and applied a Stackelberg game model to explore the optimal transformation policy that can balance such conflict of interests. By comparing the effects of two forms of subsidy in China, the results first theoretically verified the importance of subsidy in stimulating the private supply of elderly care, and then emphasized that the size of the gap between supply and demand is the fulcrum of differentiated subsidy, which determines the optimal policy for the development of the elderly care market (ECM) in each aging region. Additionally, the study showed that in the process of market-oriented transformation, the government’s positive response to the demands and preferences of the public, the establishment of market supervision measures, and the increase in the elderly’s affordability all play important roles in improving social welfare. These findings not only have policy implications for the marketization of elderly care in China, but also provide meaningful references for other developing countries in the word that are experiencing or about to experience elderly care problems.

## 1. Introduction

With the increasing longevity and decreasing fertility rate, providing sustainable care for the elderly has become one of the emerging key policy issues of our time [1]. In particular, the demand of elderly care in developing countries is growing at a rate that far exceeds that experienced in their developed counterparts—a trend likely to further increase and intensify in future years [2]. It is reported that by 2050, nearly 80% of the world’s elderly will live in developing countries, with 70% of them over the age of 80 [3]; furthermore, for an individual, the likelihood of needing care by the age of 65 exceeds 35% and increases exponentially from the age of 80 [4,5]. Faced with such a pressing trend, these developing countries have to seek new solutions within the limited and imperfect welfare system to meet the huge care demands of the elderly and to maintain the sustainability of care, including its availability and affordability [6].

Traditionally, there are two main bodies that finance and provide care services for the elderly: the family and the state [7]. However, the role of the family in shouldering the responsibilities and burdens of elderly care is eroding due to socioeconomic changes like: the miniaturization of family structure [8], the mobility of children [9], the change of family concept [10], and the participation of women in labor [11]; all of these aggravate the contradiction between supply and demand. While caring for the elderly is indeed a national responsibility, if it is solely dependent on the state, especially in developing countries, the vast demand volumes will place a heavy financial burden on them [12]. The Chinese government is facing just such a problem. Although China has become the second largest economy in the world, its economic strength in providing adequate and professional elderly care services is still limited due to its large elderly population [9]. The rapidly aging population, coupled with “getting old before getting rich,” poses a major challenge for the Chinese government to address the problem of elderly care [13,14].

Faced with the huge gap between supply and demand, policymakers point new solutions to the third party on the supply side, namely the market [15]. In fact, the marketization of elderly care services has been well developed in western industrialized countries. In England, 74% of the providers of elderly care are reported to be for-profit firms [16]. In the context of China’s supply-side reform, the privatization of elderly care provision is also especially advocated by the government [17]. In recent years, some investors, including foreign firms, have been inspired and involved in the elderly care market (ECM) in China [18]. Although most of them invest in pilot projects that have not yet been scaled up, they act as seeds for the private transformation of providers of elderly care in China. The importance of rethinking providers of care for the elderly has been recognized by the government in developing countries, but the market-oriented transformation on the supply side is not easy. Encouraging the private sector to actively participate in the provision of elderly care is still a new endeavor in these countries, fraught with uncertainties and challenges [18].

One of the core issues is the conflict of interests between private investors and the government in the process of transformation. Private investors’ decision-making is often based on the measurement of economic benefits, and it is necessary to ensure the availability of profits before deciding whether to invest. The high capital investment, long payback period, and market instability of elderly care projects hinder the investors’ investment decisions in the ECM [6,18]. The government’s decision-making is often based on the improvement of social welfare, and it is necessary to take into account public demands and policy costs in the selection and adjustment of the optimal policy. Faced with the increasing demands of elderly care and the resulting social pressures, the government has to sacrifice a portion of its fiscal revenue for necessary support policies, such as subsidies and tax incentives, to encourage the investors’ participation [6,18]. However, this conflict between private and public interests does not mean that a win-win situation cannot be achieved. Studies have shown that, with the support and guidance of government policies, a sustainable care system that balances private and public interests can be established [19,20].

To promote the marketization of elderly care and make its transformation to sustainability, the government needs to choose effective policies to implement and strengthen on the basis of considering private and public interests [21]. However, most of the existing studies on market-oriented transformation policy only focus on the interests of one side. From the perspective of private interests, it is suggested to provide long-term care insurance (LTCI) or subsidies for the elderly to increase their purchasing power, and enact incentive measures for the investors to guarantee their interests [22,23,24]. From the perspective of public interests, the emphasis is placed on evaluating the efficiency of service provision and the effectiveness of policy influence on the behavior of investors [25,26,27]. Moreover, the market is a dynamic combination of supply and demand, and the choice of effective policies cannot ignore their relationship. From the perspective of social welfare maximization, this study considered private and public interests and explored the optimal policy for the market-oriented transformation of elderly care under different supply and demand gaps by comparing the effects of different policies.

Taking the specific situation of China as an example, the Stackelberg game was established to realize the purpose of the research. It is an actional game model, and deals with hierarchical game-theoretic optimization problems [28]. Previous studies have provided a rich experience for its application. Some scholars have used it to solve the problem of government intervention in the transformation from traditional industries to emerging industries and analyze the impact of policy measures on social welfare [29,30,31]. This method also has many extension models, such as the combination of the real option model, to seek the equilibrium strategy in a dynamic environment [32]. The model of the Stackelberg game was again applied in our study to analyze the optimal transformation policy for the marketization of elderly care in a dynamic supply and demand environment, an issue that to date has received little yet increasing attention in the field of sustainability transformation. Most studies on transformation have focused on the energy and environment sectors, but sustainability threats clearly emphasize the need for a transformation in health care [19].

Following the discussion of elderly care in China, including the vast unmet demand, the regional disparity of marketization, and the policy dilemma in the conflict of interests, the Stackelberg game model considering the overall welfare of the society, namely the interests of the private and public sectors, was introduced. Then, the model results of China’s two subsidy cases were presented, including the optimal policy choice for the government under different supply and demand gaps and the main factors that influence the decision of stakeholders. Finally, the specific policy implications of the results in China, along with the conclusions and limitations of the study, were included.

## 2. Elderly Care in China

### 2.1. A Vast Unmet Demand

China is facing the pressure of elderly care demands caused by the increasing of the aged population. In 2017, nearly one in four elderly adults in the world lived in China, and the proportion of the population aged 80 and over is projected to increase from 1.6% in 2015 to 11% in 2060 [18]. Furthermore, it is reported that the total number of elderly people with limitations in activities of daily living (ADL) and instrumental ADL in China will increase dramatically, from 25 million in 2008 to 113 million in 2050 [9]. Although the “Law of the People’s Republic of China on the Protection of Rights and Interests of the Aged” has officially formalized the basic obligations of family support for the elderly, the expansion of urbanization and mass migration make it difficult for young adults to balance work and the care for their older parents, resulting in an increasing number of empty nesters in both urban and rural areas [8,9]. It is worth mentioning that China’s one child policy also weakens the family’s ability to care for its older members [33].

**Figure 1:**
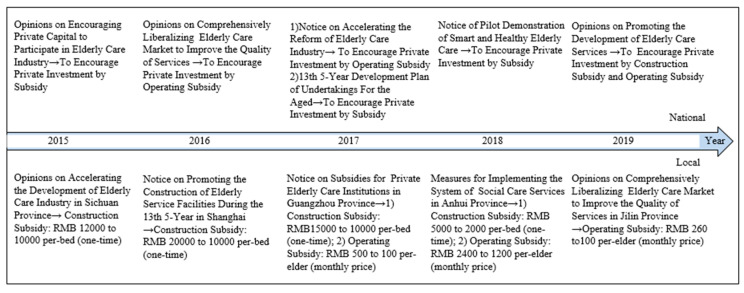
National and local governments’ incentives for private investors in the ECM in China [34,35,36,37,38,39].

To cope with the pressing demands of elderly care, the Chinese government turned to support and cultivated the ECM. As shown in Figure 1, to stimulate the market force in the provision of elderly care services, a series of favorable policies have been implemented by Chinese national and local governments in recent years [34,35,36,37,38,39]. The most important measure is the direct incentive to the investors, which includes two parts: construction subsidy (one-time subsidy on a per-bed basis) and operating subsidy (price subsidy on a per-elder basis) [17,34,35,36,37,38,39]. However, many of the specific policy reforms are made primarily by local governments to deal with the elderly care demands in their own regions, and the subsidy standards in different regions vary greatly [34,35,36,37,38,39,40]. Rational investors undoubtedly choose the regions that can obtain more profits and subsidies to invest in the ECM, which leads to the regional disparity of market-oriented transformation.

### 2.2. Regional Disparity in Marketization

The reason for variations in the subsidy standards of different regions is that the subsidy funds mainly come from local governments, and there are variations in their financial revenue [40]. In addition to the data in Figure 1, as far as we know, the highest level of construction subsidy in Beijing is RMB 16,000 per bed, yet in some regions with a low economic level, it is RMB 1000 per bed; similarly, for the operating subsidy, the monthly price compensation varies from RMB 900 to 50 per elder in different areas [40]. In the context of these policies, most of the key pilot projects are carried out in economically developed cities, such as Beijing Shuang Jin Golden Heights, Shanghai Cherish Yearn, Tianjin Hong Tai Friendship House, Fuzhou Jin Tai Yang Community Care Center, and Shenzhen Hua Ling Lian Hua North Day Care Center [18]. By contrast, some less developed regions do not have enough financial resources to provide attractive incentives for investors to establish a local care market for the elderly, because without financial support from the central government, excessive subsidies to the investors will impose more burdens on local governments.

The disparity of regional development in the market-oriented transformation of elderly care will also raise new problems of inequality and population mobility [8,9]. The central government should consider overall social welfare to ensure the sustainability of the transformation, not just the policy advocacy and approval of the marketization. Some studies have shown that without the central government’s support, China will not be able to establish a sustainable elderly care system [40]. Therefore, when the central government needs to provide financial support for local governments to develop the ECM in the top-level design of policies, it should first understand the subsidy policies of local governments according to the supply and demand situation of elderly care in different regions, and then, on the basis of private and public interests, select an effective and appropriate policy to give targeted support, instead of making transfer payments directly and aimlessly. Only in this way can the central government ensure the sustainability of the transformation and the balanced development of the ECM in different regions.

### 2.3. Conflict of Interests among Stakeholders

**Figure 2:**
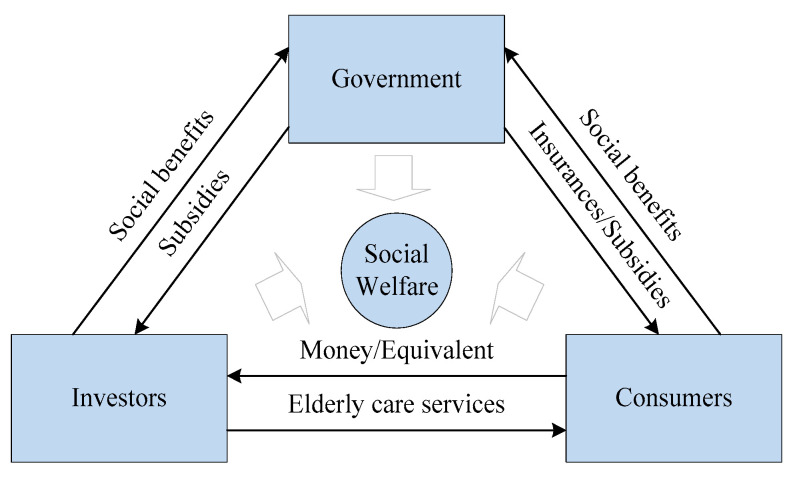
Relationships among stakeholders in the ECM.

Ideally, under policy incentives, the stakeholders and their relationships in the market-oriented transformation process of elderly care should be as shown in Figure 2. Firstly, the government anticipates the investors’ responses and designs incentives to attract their participation. Secondly, the investors are encouraged to invest in the ECM only if the reserved profit (the profit of another alternative investment) could be obtained (at least). Thirdly, the consumers (partly induced by insurances or subsidies) purchase elderly care from the investors and enjoy their professional services. In the end, each stakeholder gets what he wants, and overall social welfare is improved. However, there is a conflict of interests between the government and the investors in this process. Through subsidizing the investors, the government aims to stimulate their participation and meet public demands without incurring unnecessary policy costs, which means that the government expects to use less policy costs to obtain more social benefits. However, rational investors are profit-driven and prefer favorable policies, so they will make their investment decisions after observing the government’s policy strategies and market conditions.

When policy incentives are inappropriate and market development is immature, the investors will adopt a wait-and-see attitude instead of considering public interests to choose to immediately participate in investment in the ECM. The conflict between private and public interests undoubtedly leads to the delayed participation or hesitation of the investors, which hinders the progress of the marketization of elderly care. How to balance the interests of both sides is the key problem for the government’s policy optimization and the realization of marketization. Therefore, to explore the optimal subsidy strategy for the ECM from the perspective of social welfare maximization, it is necessary to conduct a quantitative analysis on the conflict of interest relationship, especially to discuss the strategic choice under different supply and demand gaps. After all, supply and demand determine the interests of the stakeholders.

The Stackelberg game is a kind of asymmetrical dynamic game in which the two players, the leader and the follower, have priority in making decisions [28]. The leader commits to a strategy first, knowing that the follower will take this strategy into account and react optimally [41,42]. The interaction between the government and the investors in the ECM described above is just like the process of the Stackelberg game: the government is the leader and the investors are the followers. Therefore, based on the strategic interaction model of the Stackelberg game, this study constructed a dynamic game process between the government and the investors. In the model, we first solved the investors’ optimal response to the government’s subsidies and the consumers’ demands, and then embedded this response into the government’s problem in deriving the optimal subsidy strategy under different supply and demand gaps.

## 3. Methodology

### 3.1. Notations and Assumptions

#### 3.1.1. Supply and Demand

In general, the supply means the extent to which public demands are satisfied, and the demand means the market space for the investors to profit. In other words, to a certain extent, the supply determines public interests and the demand determines private interests. Therefore, the incomes, costs, and profits of stakeholders are all affected by the supply and demand entering the market, and it is necessary to define the functions of supply and demand as well as their relationship. To simplify the exposition and unless stated otherwise, this study referred to previous studies and assumed that the market demand D is a constant elasticity function [43]:

(1) $$D\left(p,\mu \right)=Ap^{-\delta }\ln\mu$$

where A(A>0) is the scaling parameter that denotes the market size, p is the market price that ranges from $p_{0}$ to $p_{2}$, as shown in the Appendix A, δ(δ>1) is the elasticity of demand, and μ(μ>1) is the consumers’ preference, which is often affected by economic conditions, social status, publicity, and traditional filial piety culture [44,45].

In a completely market-oriented environment, supply is usually a function of price. However, in the ECM, the social role of elderly care services and the imperfect market mechanism in an early stage of development make it difficult to attract private supply through prices [24,27]. Many studies have shown that whether the investors choose to invest in the ECM is often influenced by the scale of demand and policy support [23,27,40]. If there is sufficient effective demand in the market, the investors may gain more benefits by expanding supply; if the effective demand in the market is insufficient, they may reduce the scale of investment. That is to say, the investors seeking to maximize returns will adjust the scale of supply according to demand. Therefore, the supply Q in the ECM can be a function of demand, which is expressed as:

(2) Q=αD(p,μ)

where α(α>0) is the dynamic adjustment coefficient of supply and demand. The equation contains various relationships between supply and demand, such as oversupply, overdemand, and equilibrium. It is consistent with the actual situation, where the relationship between supply and demand is constantly changing in different market environments.

#### 3.1.2. Incomes of Stakeholders

In the ECM, the incomes of stakeholders include three aspects: the economic incomes of the investors, the surplus of the consumers, and the social benefits of the government. Firstly, before making a decision, the investors often assess the investment environment of the market, especially the scale of demand, to decide whether to invest. Therefore, the expected economic incomes of the investors $R_{e}$ depend on the price and demand in the ECM, which is consistent with the definition of income function in the existing studies [29,30]. The function of $R_{e}$ can be expressed as:

(3) $$R_{e}=pD\left(p,\mu \right)$$

Secondly, similar to the studies of [30] and [43], the expected surplus of the consumers $R_{c}$ is assumed to be the difference between the total amount that customers are willing and able to pay for elderly care services and the total amount that they actually pay, which can be expressed as:

(4) $$R_{c}=\left(\theta p_{c(\xi ,\varphi )}-p\right)D\left(p,\mu \right)$$

where $p_{c(\xi ,\varphi )}$ represents the ability to pay or purchasing power of the consumers, which is positively related to the insurances ξ or subsidies φ they received, and θ(θ>0) is introduced to explain that the consumers’ willingness to pay may be heterogeneous across individuals.

Thirdly, because the expected social benefits of the government $R_{s}$ mean public well-being and social stability, $R_{s}$ should also be related to the demand, and can be expressed as:

(5) $$R_{s}=\lambda WD\left(p,\mu \right)$$

where W is assumed to be the unit social benefit, and λ(λ>1) is the parameter of public concern that reflects the urgency of public demands of elderly care, which is used to measure positive externalities of the ECM. The equation describes that the starting point of government policy is to respond to the huge care demands of the elderly. Therefore, before implementing targeted policies, the government needs to fully understand the market, especially the demands of different regions, to ensure the realization of social benefits.

#### 3.1.3. Costs of Stakeholders

In the ECM, the costs of stakeholders include two aspects: the investment costs of the investors and the policy costs of the government. The following content abstracts these costs into concrete functional expressions.

Firstly, this study considered that the investment costs of the investors are involved in the three stages of elderly care projects: construction, operation, and maintenance. Therefore, the investment costs are described quantitatively according to these stages. The construction cost $C_{c}$ is expressed as:

(6) $$C_{c}=\rho_{\left(j,k\right)}QI_{c}$$

where $I_{c}$ is the unit construction cost, and $\rho_{\left(j,k\right)}$ is the parameter of the premium cost associated with the market positioning j and expected quality level k. The operating cost $C_{p}$ is expressed as:

(7) $$C_{p}=\rho_{\left(j,k\right)}QI_{p}$$

where $I_{p}$ is the unit operating cost. The daily maintenance cost $C_{n}$ is expressed as:

(8) $$C_{n}=r\left(C_{c}+C_{p}\right)$$

where r is the maintenance rate of buildings and facilities.

Secondly, as mentioned above, because the subsidies for investors in the ECM have two parts, their policy costs G should be described respectively. Since the construction subsidy is a one-time subsidy based on the number of beds, its policy cost $G_{c}$ is associated with Q, which can be given as:

(9) $$G_{c}=m_{\left(h\right)}g_{c}Q$$

where $g_{c}$ is the unit subsidy on a per-bed basis, and $m_{\left(h\right)}\left(0\leq m_{\left(h\right)}\leq 1\right)$ is the adjustment coefficient that is influenced by the government’s quality supervision h. Since the operating subsidy is the price subsidy based on the number of consumers, its policy cost $G_{p}$ is associated with D, which can be given as:

(10) $$G_{p}=m_{\left(h\right)}g_{p}D$$

where $g_{p}$ is the unit subsidy that the investors can obtain from caring for one elder.

#### 3.1.4. Profits of Stakeholders

The expected net income of the investors $L_{e}$, namely the profit, can be given as:

(11) $$L_{e}=R_{e}-\left(C_{c}+C_{p}\right)\eta_{\left(i,T\right)}-C_{n}+G$$

where $\eta_{\left(i,T\right)}$ is the discount indicator function for interest rate i and full life cycle T.

The expected net benefit of the government $S_{w}$, namely the social welfare, can be written as:

(12) $$S_{w}=L_{e}+R_{c}+R_{s}-G$$

Other variables are the same as above.

### 3.2. Schematic Model of the Stackelberg Game

**Figure 3:**
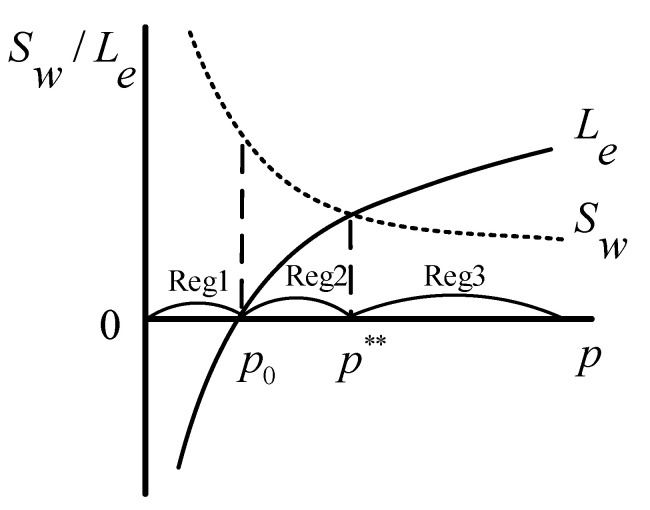
Schematic model of the Stackelberg game.

Based on Equations (11) and (12), the schematic model of the Stackelberg game is presented in Figure 3, in which $p_{0}$ is the price when G=0 and $L_{e}=0$, and $p_{}^{**}$ is the intersection of the two curves of $L_{e}$ and $S_{w}$. As shown, there exist three regions (Reg) in the game process:

Reg1: $p<p_{0}$, the investors cannot profit from the ECM, and the government does not provide any favorable policies.

In Reg1, there is no profit for the investors and only potentially high unrealized social welfare for the government. Some economic and social conditions, such as insufficient market recognition and imperfect market rules, lead to the low price in Reg1. Obviously, it is inefficient for the investors to invest in the ECM in this case. Therefore, rational investors will not choose the ECM in this region, which will result in the lack of adequate and professional care services for the elderly, and thereby the high social welfare will not be achieved.

Reg2: $p_{0}<p<p^{**}$, the government’s subsidies are provided for the investors, so that they begin to profit from the ECM.

Faced with public demands, the government has to take some measures. Reg2 is the game space in which the government formulates subsidies to cultivate the ECM and inspires the investors’ participation by sacrificing some social welfare to increase private supply. The investors get the subsidies and begin to obtain profits from the ECM. However, the government also cares about policy costs, and its game strategy will make the policy costs as small as possible, especially not greater than the gains. This study especially focused on this region to seek an effective and appropriate policy under different supply and demand gaps.

Reg3: $p^{**}<p$, the investors’ profits increase even without any favorable policies from the government.

In Reg3, under the cultivation of previous policies, the market has developed and the price has gradually increased. In this case, even if there are no incentives, the investors can get more profits. That is to say, in Reg3, the ECM is self-inducible, and the investors will invest in it spontaneously. Therefore, as the game leader, the government will not make any favorable policies because of their huge costs. This region reflects the free development of the ECM, which is also the ultimate goal of the government.

## 4. Results

### 4.1. Optimal Subsidy Choice of the Government

In the game, there is always a conflict of interests between players. The process of the game is the process in which participants seek their own equilibrium strategy in the constant interaction of conflict and adjustment. In Reg2, the interaction between the investors pursuing private interests and the government representing public interests is such a process. This subsection specially analyzed Reg2 to explore the optimal policy choice for the government by examining the effects of different subsidy forms on the equilibrium strategy. Considering that the subsidies consist of two parts, we divided the game process between the government and the investors into two cases to compare their policy effects.

**Case 1.** Construction subsidy:

Following this policy, the profits of the investors are

(13) $$\begin{array}{l} L_{e1}^{}=R_{e}-\left(C_{c}+C_{p}\right)\eta_{\left(i,T\right)}-C_{n}+G_{c}\\ \;\;=D\left(p,\mu \right)\left(p-\alpha \left(\rho_{\left(j,k\right)}\left(I_{c}+I_{p}\right)\left(\eta_{\left(i,T\right)}+r\right)-m_{\left(h\right)}g_{c}\right)\right) \end{array}$$

The critical pair of $\left\{p_{1}^{*},D_{1}^{*}\right\}$ can be obtained as (see Appendix A):

(14) $$\left\{ \begin{array}{l} p_{1}^{*}=\alpha \delta {\left(\delta -1\right)}^{-1}\left(\rho_{\left(j,k\right)}\left(I_{c}+I_{p}\right)\left(\eta_{\left(i,T\right)}+r\right)-m_{\left(h\right)}g_{c}\right)\\ D_{1}^{*}=A\ln\mu \cdot {\left(\alpha \delta {\left(\delta -1\right)}^{-1}\left(\rho_{\left(j,k\right)}\left(I_{c}+I_{p}\right)\left(\eta_{\left(i,T\right)}+r\right)-m_{\left(h\right)}g_{c}\right)\right)}^{-\delta } \end{array}\right.$$

Therefore, in this case, the social welfare of the government is

(15) $$S_{w1}^{*}=D_{1}^{*}\left(\theta p_{c(\xi ,\varphi )}-\alpha \rho_{\left(j,k\right)}\left(I_{c}+I_{p}\right)\left(\eta_{\left(i,T\right)}+r\right)+\lambda W\right)$$

**Case 2.** Operating subsidy:

Following this policy, the profits of the investors are

(16) $$\begin{array}{l} L_{e2}^{}=R_{e}-\left(C_{c}+C_{p}\right)\eta_{\left(i,T\right)}-C_{n}+G_{p}\\ \;\;=D\left(p,\mu \right)\left(p-\alpha \left(\rho_{\left(j,k\right)}\left(I_{c}+I_{p}\right)\left(\eta_{\left(i,T\right)}+r\right)-\alpha^{-1}m_{\left(h\right)}g_{p}\right)\right) \end{array}$$

The critical pair of $\left\{p_{2}^{*},D_{2}^{*}\right\}$ can be obtained as (see Appendix A):

(17) $$\left\{ \begin{array}{l} p_{2}^{*}=\alpha \delta {\left(\delta -1\right)}^{-1}\left(\rho_{\left(j,k\right)}\left(I_{c}+I_{p}\right)\left(\eta_{\left(i,T\right)}+r\right)-\alpha^{-1}m_{\left(h\right)}g_{p}\right)\\ D_{2}^{*}=A\ln\mu \cdot {\left(\alpha \delta {\left(\delta -1\right)}^{-1}\left(\rho_{\left(j,k\right)}\left(I_{c}+I_{p}\right)\left(\eta_{\left(i,T\right)}+r\right)-\alpha^{-1}m_{\left(h\right)}g_{p}\right)\right)}^{-\delta } \end{array}\right.$$

Therefore, in this case, the social welfare of the government is

(18) $$S_{w2}^{*}=D_{2}^{*}\left(\theta p_{c(\xi ,\varphi )}-\alpha \rho_{\left(j,k\right)}\left(I_{c}+I_{p}\right)\left(\eta_{\left(i,T\right)}+r\right)+\lambda W\right)$$

**Remark** **1.**  *In both cases, the investors’ profits increase, which suggests that the subsidies can change the investors’ choice. In reality, if the subsidies were high enough, many unprofitable projects would become profitable, even if the price was much lower. However, the government has to consider policy costs and the corresponding loss of social welfare, so that it needs to choose an optimal policy strategy for the ECM. By comparing Equations (13)–(18), intuitively speaking, the main differences between the optimal results in Case 1 and Case 2 are*$g_{c}$*and*$\alpha^{-1}g_{p}$*. That is to say, the parameter of*α*, the unit subsidy of*$g_{c}$*, and*$g_{p}$*play important roles in causing the difference of the profits*$L_{e}^{}$*, the optimal price*$p_{}^{*}$, demand$D_{}^{*}$, and social welfare$S_{w}^{*}$in the two cases. Therefore, before seeking an optimal subsidy policy, the government should first have a clear grasp of theα, $g_{c}$*, and*$g_{p}$*in different regions.*

*When*$g_{c}>\alpha^{-1}g_{p}$*, that is,*$\alpha >g_{p}/g_{c}$*, we can easily get*$L_{e1}>L_{e2}$*,*$D_{1}^{*}>D_{2}^{*}$, and$p_{1}^{*}<p_{2}^{*}$, which means that the construction subsidy in Case 1 can bring more profits and greater actual demand for the investors, and lower market price for the consumers. Moreover, the inequality of$D_{1}^{*}>D_{2}^{*}$,$S_{w1}^{*}>S_{w2}^{*}$can be obtained based on Equations (15) and (18). In this case, compared to the operating subsidy in the later stage of the project, the construction subsidy in the early stage is more conducive to stimulating the enthusiasm of private supply, increasing the effective market demand, and improving the social welfare. Similarly, when$g_{c}<\alpha^{-1}g_{p}$*, that is,*$\alpha <g_{p}/g_{c}$*, we can derive that the operating subsidy in Case 2 is more effective in achieving the above goals. When*$g_{c}=\alpha^{-1}g_{p}$*, that is,*$\alpha =g_{p}/g_{c}$*, the two forms of subsidies have no difference in policy effects. However, because the relationship between supply and demand in the market is always dynamic, such equality does not seem to exist in practice. According to Equation (2), we can obtain*α=Q/D*, which indicates*α*is affected by the values of*Q and D*. Therefore, the government’s choice of subsidy strategy in Case 1 or Case 2 depends on the gap between supply and demand that serves as the policy fulcrum.*

*Furthermore, according to Equations (15) and (18), we can further find that the social welfare* $S_{w1}^{*}$ *and* $S_{w2}^{*}$ *in the two cases are negatively related to the parameter of* α *and positively related to the variable of* $p_{c(\xi ,\varphi )}$ *. Because* α=Q/D *, with the demand* D *becoming larger, the value of* α *will be smaller. That is to say, when the demand expands, the space of social welfare improvement will also become larger. The model theoretically demonstrates the importance of the government’s support for market-oriented transformation of elderly care in improving social welfare. Due to the positive correlation between* $S_{w}^{*}$ *and* $p_{c(\xi ,\varphi )}$ *, if the government increases the levels of insurance or subsidies for the elderly to improve their real purchasing power, the social welfare will also increase accordingly.*

Finally, from an intuitive point of view, in Case 1, because the construction subsidy $G_{c}$ has a relationship with the investment scale of Q, the investors are more likely to lie to the government and expand the construction scale indiscriminately to obtain more subsidies and ignore how to operate in the later period. That is the reason why there are so many empty beds now in the ECM [46]. In Case 2, the operating subsidy $G_{p}$ is positively related to the market demand D. The investors are more likely to exaggerate their services to attract more consumers, since the number of consumers determines the amount of subsidy they can receive. Therefore, both kinds of subsidy are inseparable from the government’s regulation.

### 4.2. Influence of Parameters on Stakeholders’ Decisions

**Figure 4:**
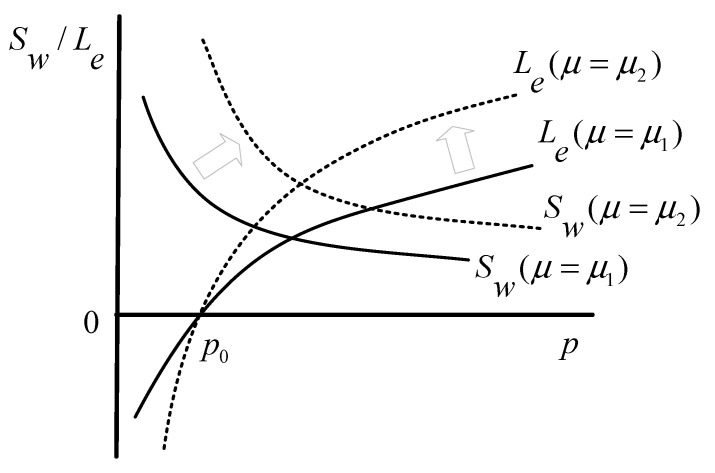
Schematic of the influence of parameter μ on the investors’ profits and social welfare ($\mu_{1}<\mu_{2}$).

**Figure 5:**
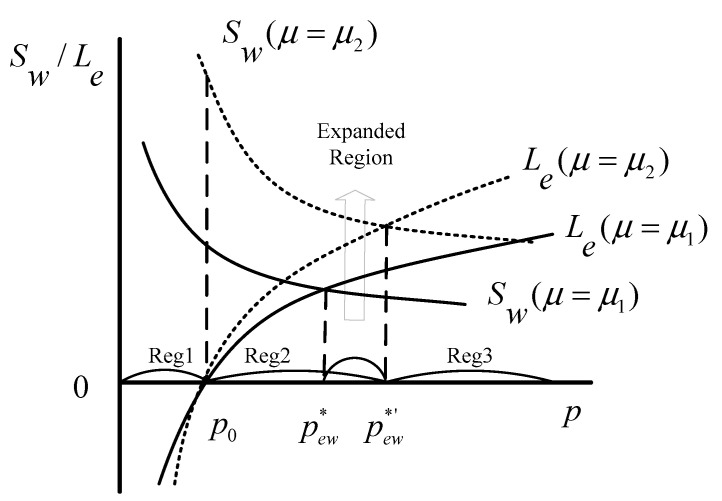
Schematic of the influence of parameter μ on the game process ($\mu_{1}<\mu_{2}$).

In addition to variables, such as benefit, cost, demand, and price, that affect the decision-making and game process of stakeholders, some parameters, such as the consumers’ preference and public concern, also have impacts. By acting on the demand function, the consumers’ preference is positively correlated with the investors’ profits and social welfare, which can be explained by Equations (11) and (12). As described in Figure 4, the larger value of μ can make both function curves of profit and social welfare move up, which indicates that the consumers’ preference can change the choices of the investors and the government in the ECM. Figure 5 shows the influence of the customers’ preference on the game process. As illustrated, the larger value of μ can expand Reg2 and narrow Reg3, but has no impact on Reg1, indicating that a higher level of the customers’ preference can promote the game between the government and the investors, which is conducive to establishing an adequate care market for the elderly.

**Figure 6:**
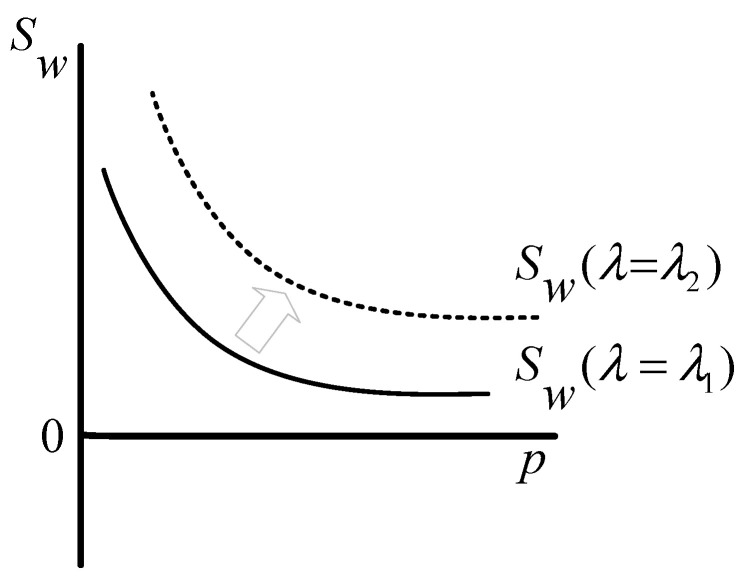
Schematic of the influence of parameter λ on the social welfare ($\lambda_{1}<\lambda_{2}$).

**Figure 7:**
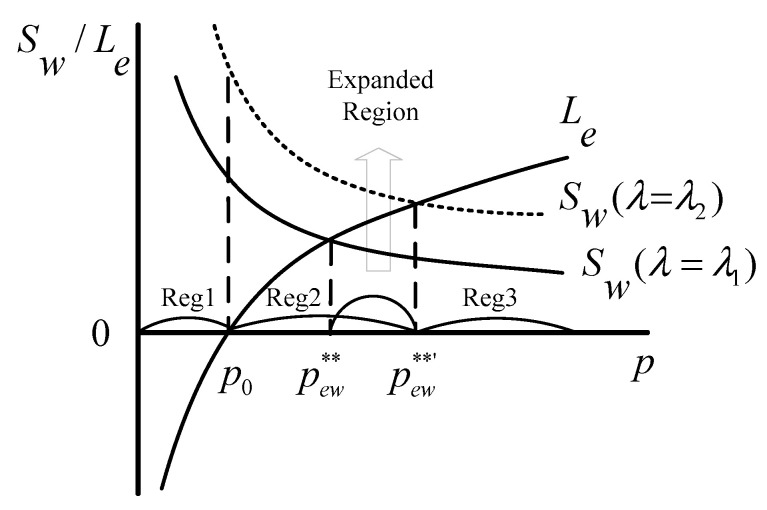
Schematic of the influence of parameter λ on the game process ($\lambda_{1}<\lambda_{2}$).

The parameter of λ is introduced to represent the public concern. According to Equation (12), the social welfare $S_{w}$ is positively related to the parameter of λ. Figure 6 clearly describes the relationship between them. As shown, the greater value of λ can make the function curve of social welfare move up, which illustrates that the higher level of public concern represents a greater growth potential for social welfare. Therefore, to some extent, the public concern can nudge the government to support and develop the ECM, to play its positive externalities. As with the consumers’ preference μ, the parameter of λ also has a significant effect on the game process. A higher level of public concern also can expand Reg2 and narrow Reg3. Figure 7 clearly shows how Reg1, Reg2, and Reg3 transform with the variation of λ. Therefore, when the market price is relatively low, a greater level of public concern is more likely to result in government intervention for the ECM.

## 5. Policy Implications

Whether it is a local or central government, the responsibility of the government forces it to respond to the public demand of elderly care. The most important way for the government to realize the development of the ECM and provide care services for the elderly is policy support [40,47,48]. This study showed the importance of providing subsidies for the investors to privatize the supply of elderly care, which is consistent with the conclusions of existing studies [40,47,48]. China is a vast country, with different economic and social development in different regions, and many policy measures need to adapt to local conditions [49]. Therefore, when the central government hopes to enhance the financial strength of local governments through transfer payments and ensure their abilities to support the marketization of elderly care, it also needs to combine the actual situation of different regions. That is to say, the central government should choose the most suitable form of subsidy to ensure the maximum effect of the policy.

According to published reports [50,51], we summarized the government’s revenue and the proportion of elderly people in provincial regions in 2017, and calculated the gap between supply and demand of elderly care α in the market of each region. To highlight regional differences and give clear results, we also calculated the ratio of demand to supply $\alpha^{-1}$. Moreover, to focus on the aging regions, we selected those with more than 10% of elderly people for analysis. As shown in Table 1, they were classified into two categories according to their respective government revenue: (I) the regions with per capita fiscal revenue above RMB 10,000, including Beijing, Tianjin, Shanghai, Jiangsu, and Zhejiang; (II) the regions with per capita fiscal revenue below RMB 10,000, including Hebei, Liaoning, Jilin, Heilongjiang, Anhui, Shandong, Henan, Hunan, Chongqing, Sichuan, Shaanxi, and Gansu. Obviously, we could see that the value of $\alpha^{-1}$ in the regions of category II is much larger than those of category I. Apart from the lack of financial resources of local governments to attract private participation, most of the regions in category II are located in the central or western areas, where the investment market itself is inactive, resulting in the gap between demand and supply being enlarged [40,52]. While the regions in category Ⅰ have a higher degree of aging, the fiscal strength of local governments is also stronger, and these regions are politically and economically active with a large number of investors, making the gap between demand and supply narrow [40,52].

Based on the results of the Stackelberg game, the central government should strengthen the subsidy policies of local governments in a more targeted manner. If, at the initial stage, the central government’s financial support makes the two forms of subsidy in Case 1 and Case 2 reach the same level in each aging region, then the following successful policy adjustment should be to increase the construction subsidy in the regions with lager values of α and increase the operating subsidy in those with smaller ones, which is conducive to giving full play to the advantage of the policy itself. In the regions of category Ⅰ, the central government’s choice to support the construction subsidy of local governments can make $g_{p}/g_{c}$ smaller, which in turn makes the inequality of $\alpha >g_{p}/g_{c}$ easily satisfied. Because the land use price is much higher in these regions, it also helps ease the initial investment pressure on the investors. Similarly, in the regions of category Ⅱ, the central government’s transfer payments to the operating subsidy can make $g_{p}/g_{c}$ larger and the inequality of $\alpha <g_{p}/g_{c}$ easily satisfied, which means that the effect of subsidy policy can be better realized. The application of the model results in Table 1 further illustrates that the selection of the optimal subsidy policy depends on the size of the gap between supply and demand.

Moreover, the consumers’ preference and public concern both affect the decision-making of stakeholders, and they play a role by influencing the demand and social benefits, respectively. Some studies have indicated that men with higher education and income are more likely to choose institutional care [53,54,55], and with the rapid aging of the population, the traditional culture of elderly care is changing from family-provided to society-provided, especially for single-child families [44]. It shows that the consumers have a certain preference for privately provided elderly care services. Although it may be affected by demographic characteristics, cultural evolution, and family structure, the potential demand space contains huge development potential [55,56]. Following this trend, the government’s support and cultivation of the ECM through policy measures can not only enhance the consumers’ confidence in the private supply market and further guide their tendency to consume the services provided by private sectors, but also can convey to the society that the government is actively responding to public concern about care for the elderly, to finally achieve the goal of improving the welfare of the whole society.

Our research also showed that to ensure the maximum social effect of transfer payments, the government needs to supervise the late operation of construction subsidy projects and the behavior of the investors in operating subsidy projects by implementing some measures, such as project tracking, result evaluation, information disclosure, and quality supervision. Some supervision measures will also create a virtuous circle for the development of the ECM [57,58]. For example, project tracking can effectively prevent the investors from blindly expanding their investment scale in pursuit of the construction subsidy, and the resulting waste of resources and business difficulties. Result evaluation can summarize the effects of subsidy policy and help reflect problems in actual policy implementation. Information disclosure can play a role in promoting the ECM to the society and strengthening the supervision mechanism among stakeholders. Quality supervision is an important means to ensure the quality efforts of self-interested investors, which not only protects the rights and interests of the consumers, but also acts on the market demand in turn, thereby affecting the long-term interests of the investors themselves [57,58].

In the game model, the consumers’ surplus is part of the social welfare pursued by the government, which explains the significance of improving the consumers’ affordability in marketization reform of elderly care. Foreign experience has shown that LTCI can increase the ability of elderly people to purchase private care services; for example, South Korea and Singapore are following the example of Japan and Germany [59,60,61]. In China, LTCI system pilots were launched by the Ministry of Human Resources and Social Security in 2016 [62]. However, it has raised new issues, such as the division of intergovernmental fiscal responsibility and sharing of the burden of private payments [40]. More importantly, China’s current basic old-age security system is not perfect. Despite the expanding coverage of old-age insurance in China, nearly 200 million persons are still not covered by any old-age insurance [63]. A study shows that the average pension in urban areas of China in 2016 is only RMB 2373 per month, resulting in more than half of the elderly in these areas in need of care unable to afford the cost of institutional care [40]. Therefore, the key to improving the elderly’s affordability in China is to establish a multi-channel and strong social security mechanism.

## 6. Conclusions

The experience of developed countries has proved that the market-oriented transformation on the supply side is a feasible and sustainable solution to cope with the large unmet care demands of the elderly [64,65]. However, this transformation in developing countries is not easy. In China, because the incentive funds for the investors mainly come from local governments, the imbalance of regional economic development also results in the regional disparity of the marketization of elderly care. Although the central government can enhance the financial strength of local governments to develop the ECM by way of transfer payments, it is necessary to clarify the most effective form of subsidy for the investors in different regions before making decisions to ensure that the driving effect of a large amount of financial support is suitable for balanced development of the ECM. From the perspective of social welfare maximization, our research considered the interest relations of multiple subjects, namely private and public interests, and introduced the dynamic changes of regional supply and demand into the model, which is a supplement and improvement to the current single-agent and static incentive policy studies. Moreover, the generalized model based on actual problems in our study is more applicable to different supply and demand gaps.

Specifically, based on the marketization process and policy background of elderly care in China, we established a Stackelberg game model that considered the interests of multiple players. The social benefits of the government, the economic profits of the investors, and the surplus of consumers are integrated into the welfare of the whole society, and the optimal policy is sought on this basis. By comparing policy effects under the two subsidy cases in China, the research results theoretically verified the important role of subsidies in stimulating the investors’ participation, and emphasized the effect of the size of the gap between supply and demand on optimal subsidy strategy. According to these results, combined with the financial situation of local governments and the aging trend in various regions of China, we put forward concrete suggestions on which subsidy policy the central government should choose to support local governments to develop the ECM in different regions. Our study also showed that, in the process of market-oriented transformation, the government’s practical response to the demands and preferences of the public, the establishment of market supervision measures, and the increase in the elderly’s affordability all have important impacts on the improvement of social welfare.

Longevity is a symbol of human progress and an opportunity for social innovation. By presenting the experience of the market-oriented transformation of elderly care in China, this study aimed to provide meaningful references for other developing countries in the world that are experiencing or about to experience the problem of elderly care caused by the rapid aging of the population. There are also several limitations in the study that need to be further improved in the future. Firstly, the consumers in different regions may have different preferences for the services in the ECM; this study did not consider this differentiation when analyzing the effect of the consumers’ preference on the decision-making of stakeholders. Secondly, the balanced development of the ECM in urban and rural areas is related to the fairness and accessibility of elderly care services; this study did not collect more micro-data and analyze it in combination with the household registration system. Thirdly, the asymmetric information about investors’ motivation and the quality of care services in the ECM may lead the investors to lie to the government and lead the consumers to hesitate to use the services; this study did not analyze the influences and countermeasures of these aspects in detail.

## Figures and Tables

**Table 1:** Classification based on regional economic conditions [50,51].

Region	Per Capita Government Revenue (RMB)	Age 65+ (% of Population)	The Gap between Supply and Demand		Category
			$\alpha^{-1}=D/Q$	α=Q/D	
Beijing	25015	12.50%	14.74	0.068	Ⅰ
Tianjin	14839	11.29%	27.61	0.036	
Shanghai	27470	14.26%	21.42	0.047	
Jiangsu	10178	13.93%	22.47	0.045	
Zhejiang	10261	12.48%	20.05	0.050	
Hebei	4300	11.80%	40.47	0.025	Ⅱ
Liaoning	5477	14.08%	30.33	0.033	
Jilin	4457	12.20%	30.38	0.033	
Heilongjiang	3281	12.14%	29.92	0.033	
Anhui	4496	13.00%	34.83	0.029	
Shandong	6095	12.94%	34.55	0.029	
Henan	3564	10.84%	66.73	0.015	
Hunan	4020	12.17%	48.51	0.021	
Chongqing	7325	14.28%	41.68	0.024	
Sichuan	4310	13.93%	33.92	0.029	
Shaanxi	5233	11.10%	40.47	0.025	
Gansu	3106	10.33%	98.11	0.010	

## Appendix A

When no subsidies are provided, the profits for the investors are $L_{e}^{\prime }=R_{e}-\left(C_{c}+C_{p}\right)\eta_{\left(i,T\right)}-C_{n}$. To get the maximum value, $L_{e}^{\prime }$ is required to satisfy the first-order condition $dL_{e}^{\prime }/dp=0$ and the second-order condition $d^{2}L_{e}^{\prime }/dp^{2}<0$.

The first-order condition is derived as

(A1) $$Ap^{-\delta }\ln\mu -\delta Ap^{-\delta -1}\ln\mu \cdot \left(p-\alpha \rho_{\left(j,k\right)}\left(I_{c}+I_{p}\right)\left(\eta_{\left(i,T\right)}+r\right)\right)=0$$

and $p_{1}=\alpha \rho_{\left(j,k\right)}\delta {\left(\delta -1\right)}^{-1}\left(I_{c}+I_{p}\right)\left(\eta_{\left(i,T\right)}+r\right)$ is obtained.

The second-order condition is derived as

(A2) $$\delta \left(\delta +1\right)Ap^{-\delta -2}\ln\mu \cdot \left(p-\alpha \rho_{\left(j,k\right)}\left(I_{c}+I_{p}\right)\left(\eta_{\left(i,T\right)}+r\right)\right)-2\delta Ap^{-\delta -1}\ln\mu <0$$

and $p_{2}<\alpha \rho_{\left(j,k\right)}{\left(\delta -1\right)}^{-1}\left(\delta +1\right)\left(I_{c}+I_{p}\right)\left(\eta_{\left(i,T\right)}+r\right)$ is obtained.

Let $L_{e}^{\prime }=0$, then $p_{0}=\alpha \rho_{\left(j,k\right)}\left(I_{c}+I_{p}\right)\left(\eta_{\left(i,T\right)}+r\right)$ is obtained. Therefore, $p_{0}<p_{1}<p_{2}$, $p^{*}=p_{1}$.

Because $D\left(p,\mu \right)=Ap^{-\delta }\ln\mu$, the critical pair of $\left\{p^{*},D^{*}\right\}$ can be obtained as follows:

(A3) $$\left\{ \begin{array}{l} p_{}^{*}=\alpha \rho_{\left(j,k\right)}\delta {\left(\delta -1\right)}^{-1}\left(I_{c}+I_{p}\right)\left(\eta_{\left(i,T\right)}+r\right)\\ D_{}^{*}=A\ln\mu \cdot {\left(\alpha \rho_{\left(j,k\right)}\delta {\left(\delta -1\right)}^{-1}\left(I_{c}+I_{p}\right)\left(\eta_{\left(i,T\right)}+r\right)\right)}^{-\delta } \end{array}\right.$$

In the same way, the critical pair of $\left\{p_{1}^{*},D_{1}^{*}\right\}$ in Case 1 is obtained as

(A4) $$\left\{ \begin{array}{l} p_{1}^{*}=\alpha \delta {\left(\delta -1\right)}^{-1}\left(\rho_{\left(j,k\right)}\left(I_{c}+I_{p}\right)\left(\eta_{\left(i,T\right)}+r\right)-m_{\left(h\right)}g_{c}\right)\\ D_{1}^{*}=A\ln\mu \cdot {\left(\alpha \delta {\left(\delta -1\right)}^{-1}\left(\rho_{\left(j,k\right)}\left(I_{c}+I_{p}\right)\left(\eta_{\left(i,T\right)}+r\right)-m_{\left(h\right)}g_{c}\right)\right)}^{-\delta } \end{array}\right.$$

and $\left\{p_{2}^{*},D_{2}^{*}\right\}$ in Case 2 is obtained as

(A5) $$\left\{ \begin{array}{l} p_{2}^{*}=\alpha \delta {\left(\delta -1\right)}^{-1}\left(\rho_{\left(j,k\right)}\left(I_{c}+I_{p}\right)\left(\eta_{\left(i,T\right)}+r\right)-\alpha^{-1}m_{\left(h\right)}g_{p}\right)\\ D_{2}^{*}=A\ln\mu \cdot {\left(\alpha \delta {\left(\delta -1\right)}^{-1}\left(\rho_{\left(j,k\right)}\left(I_{c}+I_{p}\right)\left(\eta_{\left(i,T\right)}+r\right)-\alpha^{-1}m_{\left(h\right)}g_{p}\right)\right)}^{-\delta } \end{array}\right.$$

## References

[1] Pekkarinen S., Melkas H. (2019). Welfare state transition in the making: Focus on the niche-regime interaction in Finnish elderly care services. Technol. Forecast. Soc. Chang..

[2] World Health Organization (2003). Key Policy Issues in Long-term Care. https://apps.who.int/iris/bitstream/handle/10665/42604/9241562250.pdf;jsessionid=E94E6F11E1ED57BCD4150253B3FADE53?sequence=1.

[3] United Nations (2017). World Population Ageing. http://www.un.org/en/development/desa/population/theme/ageing/WPA2017.shtml.

[4] Brown J.R., Finkelstein A. (2009). The private market for long-term care insurance in the United States: A review of the evidence. J. Risk Insur..

[5] Cremer H., Pestieau P. (2014). Social long-term care insurance and redistribution. Int. Tax Public Financ..

[6] Ng A.W., Leung T.C., Tsang A.K.T. (2020). Social Enterprise for Elderly Housing: Policy for Accountability and Public-Private Responsible Financing. J. Popul. Ageing.

[7] Cremer H., Gahvari F., Pestieau P. (2017). Uncertain altruism and the provision of long term care. J. Public Econ..

[8] Liu J., Tian J., Yue P., Wang Y., Du X., Chen S. (2015). Living experience and care needs of Chinese empty-nest elderly people in urban communities in Beijing, China: A qualitative study. Int. J. Nurs. Sci..

[9] Lei P., Feng Z., Wu Z. (2016). The availability and affordability of long-term care for disabled older people in China: The issues related to inequalities in social security benefits. Arch. Gerontol. Geriatr..

[10] Hanaoka C., Norton E.C. (2008). Informal and formal care for elderly persons: How adult children’s characteristics affect the use of formal care in Japan. Soc. Sci. Med..

[11] Sugawara S., Nakamura J. (2014). Can formal elderly care stimulate female labor supply? The Japanese experience. J. Jpn. Inst. Econ..

[12] Xu X., Chen L. (2019). Projection of Long-Term Care Costs in China, 2020–2050, Based on the Bayesian Quantile Regression Method. Sustainability.

[13] Zhou G., The Influence of China’s Population Aging on Economic Growth (2018). International Conference on Economics, Finance, Business, and Development. https://webofproceedings.org/proceedings_series/ECOM/ICEFBD%202018/ICEFBD092.pdf.

[14] Guo A., Ding X., Zhong F., Cheng Q., Huang C. (2019). Predicting the Future Chinese Population using Shared Socioeconomic Pathways, the Sixth National Population Census, and a PDE Model. Sustainability.

[15] Stolt R., Blomqvist P., Winblad U. (2011). Privatization of social services: Quality differences in Swedish elderly care. Soc. Sci. Med..

[16] Barron D.N., West E. (2017). The quasi-market for adult residential care in the UK: Do for-profit, not-for-profit or public sector residential care and nursing homes provide better quality care?. Soc. Sci. Med..

[17] General Office of the State Council (2013). Circular No. 35 on Accelerating the Development of the Marketization of Elderly Care. http://www.gov.cn/xxgk/pub/govpublic/mrlm/201309/t20130913_66389.html.

[18] World Bank (2018). Options for Aged Care in China: Building an Efficient and Sustainable Aged Care System. https://openknowledge.worldbank.org/handle/10986/29807.

[19] Janssen M., Moors E.H. (2013). Caring for healthcare entrepreneurs—Towards successful entrepreneurial strategies for sustainable innovations in Dutch healthcare. Technol. Forecast. Soc. Chang..

[20] Olsen I.T. (1998). Sustainability of health care: A framework for analysis. Health Policy Plan..

[21] Alkemade F., Hekkert M.P., Negro S.O. (2011). Transition policy and innovation policy: Friends or foes?. Environ. Innov. Soc. Trans..

[22] Mou H., Winer S.L. (2015). Fiscal Incidence When Family Structure Matters: The Case of Subsidization of Home Care for the Elderly. Public Financ. Rev..

[23] Kim H., Kwon S., Yoon N.H., Hyun K.R. (2013). Utilization of long-term care services under the public long-term care insurance program in Korea: Implications of a subsidy policy. Health Policy.

[24] Yasuoka M. (2018). Subsidies for elderly care with a pay-as-you-go pension. J. Econ. Ageing.

[25] Phelan A. (2015). Protecting care home residents from mistreatment and abuse: On the need for policy. Risk Manag. Healthc. Policy.

[26] Bernoth M., Dietsch E., Burmeister O.K., Schwartz M. (2014). Information management in aged care: Cases of confidentiality and elder abuse. J. Bus. Ethics..

[27] Forder J., Netten A. (2000). The price of placements in residential and nursing home care: The effects of contracts and competition. Health Econ..

[28] Von Stackelberg H. (1952). The Theory of the Market Economy.

[29] Wang K., Zhao Y., Cheng Y., Choi T.-M. (2014). Cooperation or Competition? Channel Choice for a Remanufacturing Fashion Supply Chain with Government Subsidy. Sustainability.

[30] Sinayi M., Rasti-Barzoki M. (2018). A game theoretic approach for pricing, greening, and social welfare policies in a supply chain with government intervention. J. Clean Prod..

[31] Hong Z., Chu C., Zhang L., Yu Y. (2017). Optimizing an emission trading scheme for local governments: A Stackelberg game model and hybrid algorithm. Int. J. Prod. Econ..

[32] Thijssen J.J.J., Huisman K.J.M., Kort P.M. (2012). Symmetric equilibrium strategies in game theoretic real option models. J. Math. Econ..

[33] Zhang Y., Goza F.W. (2006). Who will care for the elderly in China?: A review of the problems caused by China’s one-child policy and their potential solutions. J. Aging Stud..

[34] National Policies. http://www.gov.cn/index.htm.

[35] Local Policies (Sichuan). http://www.sc.gov.cn.

[36] Local Policies (Shanghai). http://www.shanghai.gov.cn.

[37] Local Policies (Guangdong). http://www.gd.gov.cn.

[38] Local Policies (Anhui). http://www.ah.gov.cn.

[39] Local Policies (Jilin). http://www.jl.gov.cn.

[40] Li F., Otani J. (2018). Financing elderly people’s long-term care needs: Evidence from China. Int. J. Health Plan. Manag..

[41] Lagos F., Ordóñez F., Labbé M. (2017). A branch and price algorithm for a Stackelberg Security Game. Comput. Ind. Eng..

[42] Breton M., Alj A., Haurie A. (1988). Sequential Stackelberg equilibria in two-person games. J. Optim. Theory Appl..

[43] Krass D., Nedorezov T., Ovchinnikov A. (2013). Environmental taxes and the choice of green technology. Prod. Oper. Manag..

[44] Zhang L., Ding Z., Qiu L. (2019). Old Age Care Preferences among Chinese Middle-Aged Single-Child Parents and the Related Policy Implications. J. Aging Soc. Policy..

[45] Gibler K.M., Lee E. (2005). The impact of economic, demographic, and cultural changes on preferences for independent living arrangements and seniors housing in South Korea. J. Hous. Elder..

[46] Cui S.Y., Tian Y. (2017). Development Bottleneck of Old-age Care Institution and Solution: Based on the Survey of 45 Old-age Care Institutions in Shandong Province. Chin. J. Popul. Sci..

[47] Yao C., Luo Z., Yang Y., Ping Y. (2020). Analysis on the evolution game of rural pension mechanism in Beijing-Tianjin-Hebei region. Chin. J. Syst. Sci..

[48] Yang W., He J., Fang L., Mossialos E. (2016). Financing institutional long-term care for the elderly in China: A policy evaluation of new models. Health Policy Plan..

[49] Li N., Sun L., Luo X., Kang R., Jia M. (2019). Foreign Trade Structure, Opening Degree and Economic Growth in Western China. Economies.

[50] National Bureau of Statistics of China (2018). China Statistical Yearbook. http://data.stats.gov.cn/easyquery.htm?cn=E0103.

[51] (2018). Report on the Number of Elderly Care Institution in all Provinces and Cities of China. http://www.askci.com/news/chanye/20190218/1512251141826.shtml.

[52] Zhu F. (2019). Study on the characteristics of nursing institution for the aged based on ownership nature—The investigation from nationwide nursing institution for the aged in 2016. Soft Sci. Health.

[53] Gustafson K., Baofeng H. (2014). Elderly care and the one-child policy: Concerns, expectations and preparations for elderly life in a rural Chinese township. J. Cross-Cult. Gerontol..

[54] Zhang Z., Wang H. (2016). Zhuhai elderly’s old age care preferences and determinants. Popul. J..

[55] Smith J.P., Strauss J., Zhao Y. (2014). Healthy aging in China. J. Econ. Ageing.

[56] Wang J., Wang J., Cao Y., Jia S., Wu B. (2018). Perceived empowerment, social support, and quality of life among Chinese older residents in long-term care facilities. J. Aging Health.

[57] Lim J. (2020). Factors Affecting Mistreatment of the Elderly in Long-Term Care Facilities. Healthcare.

[58] Zeng Q., Wang Q., Zhang L., Xu X. (2020). Comparison of the Measurement of Long-Term Care Costs between China and Other Countries: A Systematic Review of the Last Decade. Healthcare.

[59] Chin C.W.W., Phua K.H. (2016). Long-term care policy: Singapore’s experience. J. Aging Soc. Policy.

[60] Chon Y. (2019). The marketization of childcare and elderly care, and its results in South Korea. Int. Soc. Work.

[61] Rhee J.C., Done N., Anderson G.F. (2015). Considering long-term care insurance for middle-income countries: Comparing South Korea with Japan and Germany. Health Policy.

[62] Ministry of Human Resources and Social Security (2016). Guidance on the Implementation of Long-term Care Insurance System Pilots. http://www.gov.cn/xinwen/2016-07/08/content_5089283.htm.

[63] Hu A. (2015). China’s Social Insurance in the Twentieth Century.

[64] Suzuki K. (2001). Marketization of elderly care in Sweden. EIJS Working Paper Series 137.

[65] Anttonen A., Häikiö L. (2011). Care ‘going market’: Finnish elderly-care policies in transition. Nord. J. Soc. Res..

